# Data on vascular plant species composition along two elevation gradients in the high Andes of central Chile (33°S)

**DOI:** 10.1016/j.dib.2024.111128

**Published:** 2024-11-13

**Authors:** Mary T.K. Arroyo, Ítalo Tamburrino, Valeria Robles, Karina Robles, Loreto V. Morales

**Affiliations:** a Departamento de Ciencias Ecológicas, Facultad de Ciencias, Universidad de Chile, Santiago, Chile; b Cape Horn International Center (CHIC), Puerto Williams, Chile; c Instituto de Ecología y Biodiversidad (IEB), Concepción, Chile

**Keywords:** Southern Andes, Alpine, Permanent sampling plots, Vascular plant species, Climate change

## Abstract

The results of a survey of vascular plant species along two elevational gradients in the high Andes of central Chile (33°S) are reported. Vascular plant species were recorded in 12 and 15 plots of 1600 m^2^ set up to be permanent, distributed at intervals of 100 m elevation along two elevational gradients, one in the La Parva (2500–3600 m a.s.l.) and a second in the Valle Nevado (2300–3700 m a.s.l.) area, Metropolitan Region of Santiago. All plots were square in shape except for one that was divided into two sections due to a landslide on the slope. Plots cover subalpine vegetation above the *Kageneckia angustifolia* treeline and the entire alpine belt to the upper limit of consistent vascular plant vegetation. The presence of each species in the individual plots is given along with location data for the plots, including georeferences, altitude, and sampling dates. Images of the study area are included. Scientific names, families, life forms, and native/non-native status are provided for the 168 taxa (167 species with one represented by two subspecies) recorded. Data on temperature and relative humidity for three sites in the general study area are included. The reported plot data will be useful for detailed studies on patterns of diversity (e.g., phylogenetic diversity, functional diversity) along elevational gradients in high mountains and for monitoring the upward shift of non-native species. Importantly, the data set provides a solid basis for detecting changes in species composition under ongoing climate change in the southern Andes, an area that has received limited attention to date but is highly relevant given instrumental evidence of rapid warming.

Specifications Table

**Table:** 

Subject	Plant science
Specific subject area	*Ecology and diversity*
Type of data	Tables, Images
Data collection	Data were collected on a conventional plot basis, at regular intervals, along two elevational transects. Plots were georeferenced and staked for permanency. All plant species found in the 27 plots covering the two transects were identified in the field or based on specimens collected in the field by the authors of the paper. Complementary data on temperature and relative humidity are provided.
Data source location	Data was collected in the Andes of central Chile, located east of Santiago in the Metropolitan Region of Santiago (Province: Santiago) (Fig. 1). The La Parva gradient ran from 2500 (33°20′39.456″S 70°17′54.378″W) to 3600 m a.s.l. (33°19′7.356″S 70°15′39.75″W). The Valle Nevado gradient ranged from 2300 (33°22′12.186″S 70°17′17.364″W) to 3700 m a.s.l. (33°19′16.794″S 70°13′52.746″W). The typical vegetation of the study area is shown in Figs. 2A-C.
Data accessibility	Repository name: Mendeley Data Data identification number: DOI: 10.17632/8zvjvcyv79.1 Direct URL to data: https://data.mendeley.com/datasets/8zvjvcyv79/1
Related research article	None.

## 1 Value of the Data

• Species recorded in large plots situated at regular intervals along elevational gradients have an enormous potential to improve our understanding of species distributions, species richness patterns, and other aspects of biodiversity. This is especially so if the gradients are replicated in nearby areas.
• Permanent plots are very scarce in the southern Andes in general. They are badly needed in an area of the Andes where warming at high elevations is occurring at a faster rate than at lower elevations.
• Plots set up to be permanent provide an ideal situation to detecting the rates of introduction of non-native species.

## 2 Background

Temperature decreases rapidly along elevational gradients in high mountain areas [1]. Increasing harsh environmental conditions are expected to be associated not only with changes in species richness [2], flowering phenology [3,4], pollinator composition [3], and pollination rates [5] in the central Chilean Andes, but also with other aspects of diversity. We were motivated to collect these data to test hypotheses regarding how phylogenetic diversity and structure varies with elevation. Few large-scale plots, set up in different watersheds, are available in the southern Andes [6]. This led us to implement a set of large plots at 100 m elevational intervals replicated along two gradients. The plots can also be used to study other aspects of diversity, such as functional diversity and community structure. A second, and perhaps more important reason for setting up the plots was to provide a set of permanent plots that can be resampled in the near and distant future to assess the effect of climate change on the elevational distributions of high elevation species. Instrumental evidence points to faster warming at high elevations in the central Chile [7].

## 3 Data Description

The data set consists of 27 survey plots of 1600 m^2^, one per 100 m elevation band along two elevation gradients. Twelve plots were located in the La Parva area and 15 in the Valle Nevado area in the central Chilean Andes. We include a data file in the Supplementary material that contains five sheets. The first sheet provides elevation, exposition, georeferences for the plot corners and center, and sampling dates. The second and third sheets contain species recorded in each plot along the two elevational gradients. The fourth sheet provides the scientific names, families, life form and native/non-native status of the 168 taxa (167 species with one species represented by two subspecies) recorded in the plots. A fifth provides data on temperature and relative humidity for three sites in the general study area. Data have been uploaded in the Mendeley Data open access repository (https://data.mendeley.com/datasets/8zvjvcyv79/1) as M.T.K. Arroyo, Í. Tamburrino, V. Robles, K. Robles, L.V. Morales, Plant data for large plots sampled over two elevational gradients in the high Andes of central Chile (33°S) [8].

## 4 Experimental Design, Materials and Methods

### 4.1 Study area

**Fig. 1:**
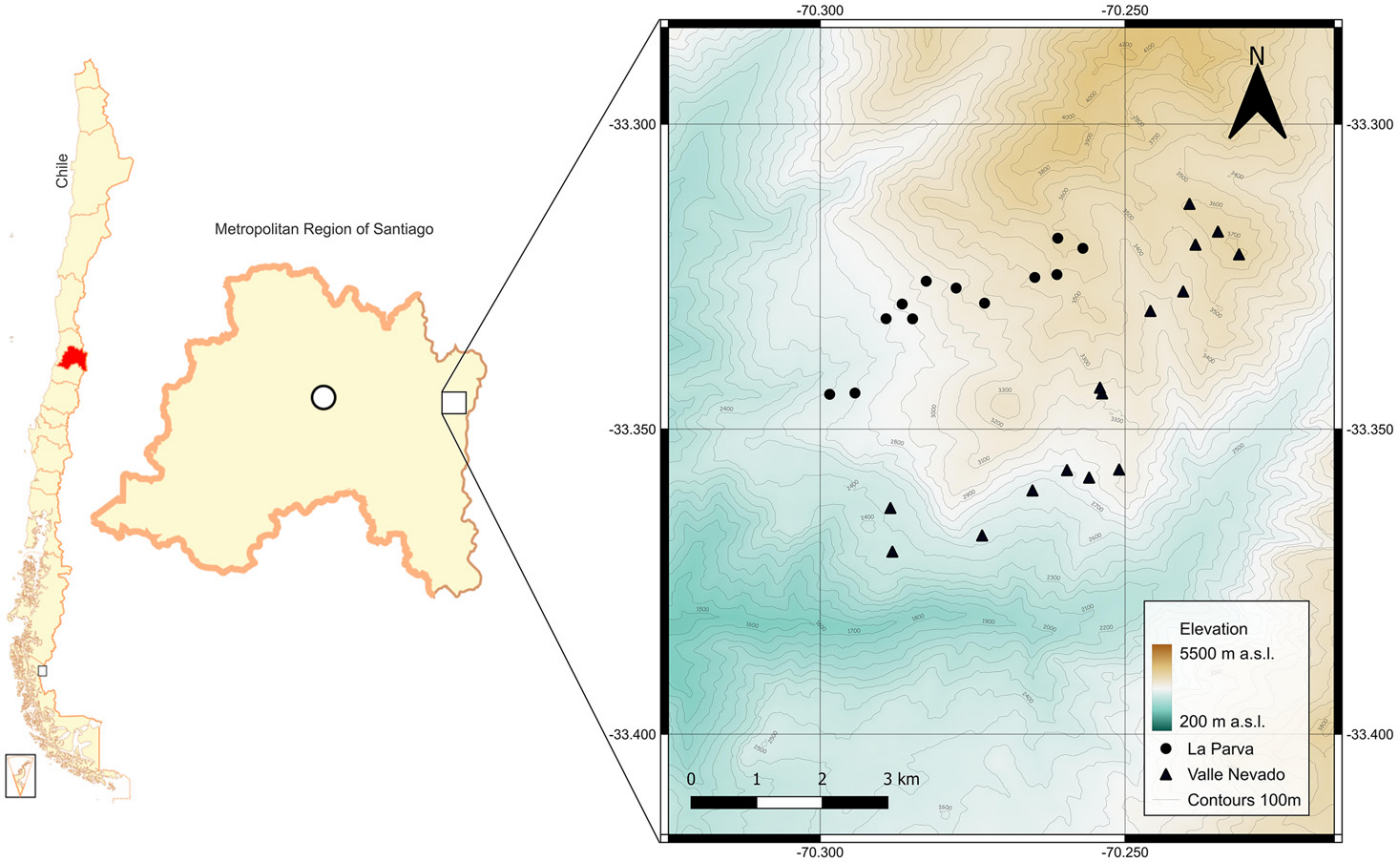
Location of the study area and individual plots. La Parva area (●); Valle Nevado area (▲).

**Fig. 2:**
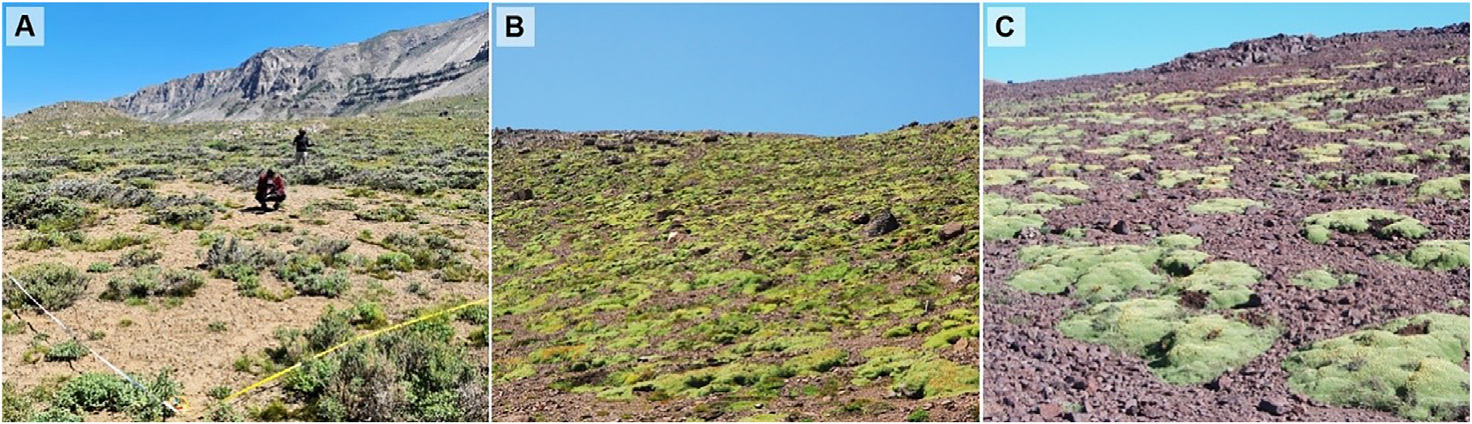
Typical vegetation in the study area. (A) Subalpine belt in the Valle Nevado area (2300 m a.s.l.) dominated by low shrubs such as *Chuquiraga oppositifolia, Acaena alpina* Poepp. ex Walp and *A. splendens* Hook. & Arn.; (B) Lower part of the alpine belt in the La Parva area (2900 m a.s.l.) dominated by the cushion- forming *Azorella ruizii* G. M. Plunkett & A. N. Nicolas; (C) Upper part of the alpine belt (in the Valle Nevado area (3600 m a.s.l.) dominated by cushion-forming *Azorella madreporica* Clos.

Data was collected in the Andes of central Chile (Fig. 1) above the *Kageneckia angustifolia* D. Don treeline to include the subalpine belt (Fig. 2A) and the entire alpine belt (Figs. 2B and C) up to the upper limit of consistent vascular plant vegetation in the La Parva and Valle Nevado areas. Subalpine vegetation in this area of the Andes is dominated by low-rounded shrubs accompanied by subshrubs, perennial, and annual herbs, while the alpine belt is dominated by cushion species and perennial herbs. The climate of the area has a strong Mediterranean influence, especially at lower elevations [9]. The growing season starts from late August to October and terminates in April, with the length of the growing season dependent on elevation and slope exposition [10,11].

### 4.2 Data collection

Plots were set up in undisturbed zonal vegetation. Azonal wetland vegetation along stream sides and alpine cushion bogs and steep scree slopes were excluded. At the height of the austral summer season, homogeneous tracts of vegetation were selected at approximately 100 m elevation intervals (hereafter, sampling sites) along the two gradients: La Parva area: 2500–3600 m a.s.l.; Valle Nevado area: 2300–3700 m a.s.l. Although some plant species occur above these elevations, they are scattered and not amenable to meaningful sampling with plots. The sampling sites are found on slopes in the vicinity of public roads up to 3000 m a.s.l. in the Valle Nevado area and 2800 m a.s.l. in the La Parva area. Above these elevations the sampled sites are found in the vicinity of non-public ski maintenance access roads. The sampling sites on the steeper (and shorter) La Parva gradient were spread out over a linear distance of 5 km. Those of the Valle Nevado gradient covered a lineal distance of 7.5 km. At each sampling site, a 40 m × 40 m (1600 m^2^) plot was laid down, with the upper and lower sides placed perpendicular to the slope. All plots were squares except for one (Valle Nevado, 2700 m a.s.l. site). Due to a small landslide on the middle of the slope, we were forced to divide this plot into two sections, one on each side of the landslide. The four corners of the plots and their center were georeferenced with a Garmin Montana 680 GPS, WGS 84 reference system. Additionally, the corners and center of each plot were fitted with metal stakes to facilitate their location in the future. The study area is a summer and winter recreational area. Consequently, the stakes had to be rammed well below ground level to avoid accidents. Using a single large plot per sampling site constitutes a large improvement regarding representing species richness over previous studies carried out in the same area (Supplementary Material: S1). Replicating the study over two gradients will be useful for detecting trends that are independent of local conditions on a given gradient.

Each plot was scrutinized for vascular plant species by three observers (including the lead author, who has worked in the area for over 40 years and has very good knowledge of the flora). This was done by walking 1 m wide slices of the plot. Field assistants recorded georeferences and put in the metal stakes. Species occurring in a series of subplots nested within each 1600 m^2^ plot were also recorded (to be published elsewhere). Depending on logistics, weather conditions, and vegetation cover, the team comprising five workers was able to sample two to six plots on a single day. Where needed, herbarium specimens were collected to ensure correct identification. Specimens were identified at the University of Concepción (CONC) herbarium and will be deposited there.

Data on average monthly temperature and relative humidity at 1.5 m a.g.l. recorded by us were available for three sites located in different elevational bands (2400, 2900, 3500 m a.s.l.) in the general study area. The data was recorded at hourly intervals with data loggers (HOBO U23 Pro v2; Onset Computer Corp., Cape Cod, MA, USA) fitted with custom-made shields (Solar Radiation Shields RS1, Onset Computer Corp., Cape Cod, MA, USA). The data pertain to the period Sept. 2019 – August 2021.

## Limitations

Although not strictly necessary for the purposes for which the data was originally obtained, it would have been useful to include a measure of plant cover. We decided against this as the plots were sampled in the latter part of a severe drought which lasted for >10 years in central Chile. While the drought would not have materially affected species richness, it is likely to have affected the abundance of species (c.f. [12]).

## Ethics Statement

The authors have read and follow the ethical requirements for publication in Data in Brief. We confirm that the current work does not involve human subjects, animal experiments, or any data collected from social media platforms.

## CRediT Author Statement

**Mary T. K. Arroyo**: Conceptualization, Methodology, Field work, Writing - Review & Editing, Funding acquisition. **Ítalo Tamburrino**: Conceptualization, Data Curation - Review & Editing. **Valeria Robles**: Field work, Data Curation. **Karina Robles**: Field work, Data Curation. **Loreto V. Morales**: Writing - Original Draft, Writing - Review & Editing, Visualization.

## Data Availability

Mendeley DataPlant data for large plots sampled over two elevational gradients in the high Andes of central Chile (33°S) (Original data).

## Appendix Supplementary materials

Image, application 1

Image, application 2
