# Supplementary material for: Data on vascular plant species composition along two elevation gradients in the high Andes of central Chile (33°S)
Source: Data Brief. 2024 Nov 13;57:111128. doi: 10.1016/j.dib.2024.111128 (PMC11617968; doi:10.1016/j.dib.2024.111128)
Supplement: Supplementary file 1 [file mmc1.docx]

# **SUPPLEMENTARY MATERIAL**

**S1. Plot size justification**

In this study, we sampled two elevational gradients. Sampling one 1600 m^2^ plot per elevational band on each of these gradients could be questioned as sufficient to represent species richness. Our 1600 m^2^ plots are very large for alpine vegetation. For example, a recent study sampled a total of one hundred 1m^2^ quadrats per site arranged along five transect lines [1], which is the equivalent of only 6.25% of the area we sampled at any one elevation. Using a single large plot per sampling site constitutes a large improvement over previous studies carried out in the same area. An early study sampled one 50 m transect every 100 m elevation on the same Valle Nevado gradient [2]. These authors recorded a total of 99 species, whereas we recorded 140 species for the same set of elevational bands. In a more recent study, between 10 and 25 species per site were reported for five elevational levels in the Valle Nevado area based on sampling 7-8 replicate 10 m transects per site [3]. For these same elevational levels, we report between 20 and 44 species in our single 1600 m^2^ plots. To our knowledge, the largest plots sampled thus far in central Chilean Andes are single 20 m x 20 m plots per elevation band [4]. For the comparable Farellones area, mean species numbers of 20.82 ± 1.69 (SE) were reported. The equivalent statistics for our plots are 36.2 ± 3.2 (SE) (La Parva transect) and 36.2 ± 3.7 (SE) (Valle Nevado transect). In another study, species richness was determined for five 2.4 m x 2.4 m subplots nested in a 20 m x 20 m plot area on 6 sites in the same general area as this study [5]. The numbers of species recorded in our single 1600 m^2^ plots for comparable elevations were consistently larger than the sum for the five 2.4 m x 2.4 m plots. Based on these comparisons, we are confident that our plots have picked very large numbers of species.

# **REFERENCES**

[1] H. [Shaheen](https://www.researchgate.net/profile/Hamayun-Shaheen?utm_content=businessCard&utm_source=publicationDetail&rgutm_meta1=AC%3A3713959&_tp=eyJjb250ZXh0Ijp7ImZpcnN0UGFnZSI6InB1YmxpY2F0aW9uIiwicGFnZSI6InB1YmxpY2F0aW9uIn19), S. Aziz, S. Nasar, R.W. Bussmann, M. Waheed, M. Manzoor, M.H. Siddiqui, S. Alamri, S. Marifatul, 2023. Distribution patterns of alpine flora for long-term monitoring of global change along a wide elevational gradient in the Western Himalayas. Glob. Ecol. Conserv. 48, e02702. <https://doi.org/10.1016/j.gecco.2023.e02702>.

[2] L.A. Cavieres, A. Peñaloza, M.T.K. Arroyo, Pisos altitudinales de vegetación en los Andes de Chile central (33°S), Rev. Chil. Hist. Nat. 73 (2000) 331 – 344. http://dx.doi.org/10.4067/S0716-078X2000000200008.

[3] L. Schroeder, V. Robles, P. Jara-Arancio, C. Lapadat, S.E. Hobbie, M.T.K. Arroyo, J. Cavender-Bares, 2024. Drivers of plant diversity, community composition, functional traits, and soil processes along an alpine gradient in the central Chilean Andes. Ecol. Evol. 14, e10888. <https://doi.org/10.1002/ece3.10888>.

[4] J. López-Angulo, D.S. Pescador, A.M. Sánchez, M.A. Mihoč, L.A. Cavieres, A. Escudero, 2018. Determinants of high mountain plant diversity in the Chilean Andes: From regional to local spatial scales. PLoS One. 13(7), e0200216. <https://doi.org/10.1371/journal.pone.0200216>.

[5] J. López-Angulo, D.S. Pescador, A.M. Sánchez, A.L. Luzuriaga, L.A. Cavieres, A. Escudero, 2019. Alpine vegetation dataset from three contrasting mountain ranges differing in climate and evolutionary history. Data in Brief. 27, 104816. https://doi.org/10.1016/j.dib.2019.104816.
